# Staff Barriers to Fulfilling Assisted Living Resident Daily Care and Activity Preferences

**DOI:** 10.1093/geroni/igaa057.3066

**Published:** 2020-12-16

**Authors:** Tonya Roberts, Jillian Parks, Ella Greenhalgh, Josephine Hansen, Olivia Wheelis, Sara Wilke

**Affiliations:** 1 University of Wisconsin-Madison, Madison, Wisconsin, United States; 2 University of Wisconsin, Madison, Wisconsin, United States

## Abstract

Person-centered care (PCC), or delivery of care consistent with preferences, has been associated with improved care and quality of life for residents in long-term care (LTC). However, research has shown PCC has not been universally adopted. While general implementation barriers have been identified, little research has focused on barriers to meeting specific types of resident daily care and activity preferences. The purpose of this study was to describe LTC staff barriers to fulfilling specific types of resident preferences. A descriptive, qualitative study with 19 assisted living staff from nursing, dietary, and activities was conducted. Semi-structured interviews focused on identifying work system barriers to meeting specific types of resident preferences were analyzed using thematic analysis. Findings suggest shift assignments, staffing challenges, and facility schedules influence staff ability to meet certain types of preferences. The results suggest innovative design of shift schedules and assignments may help staff meet certain types of preferences. Part of a symposium sponsored by the Research in Quality of Care Interest Group.

